# Case Report: Direct anterior approach with surgical hip dislocation for management of juvenile femoral head chondroblastoma: a case series and systematic review of the literature

**DOI:** 10.3389/fped.2025.1738552

**Published:** 2026-01-21

**Authors:** Xiujiang Yang, Xiaolin Luo, Xiudong Li, Ke Pang, Yuanhan Zou, Xiaofei Ding, Shijie Liao

**Affiliations:** The First Affiliated Hospital of Guangxi Medical University, Nanning, China

**Keywords:** avascular necrosis, chondroblastoma of the femoral head, direct anterior approach (DAA), pediatric bone tumors, surgical hip dislocation (SHD)

## Abstract

**Background:**

Femoral head chondroblastoma is a rare benign tumor in adolescents (10–20 years). Traditional surgeries face difficulties like poor exposure, high trauma, and risks of physeal injury/avascular necrosis (AVN). The DAA-SHD approach (no greater trochanteric osteotomy) is proposed for direct tumor resection, vascular preservation, and articular cartilage repair.

**Methods:**

A literature review (2005–2025) on adolescent femoral head chondroblastoma was conducted. Retrospective analysis of 4 cases (2014–2025) treated with supine DAA-SHD (same senior surgeon) autologous iliac bone grafting. Hip function was assessed via MSTS scale.

**Results:**

Mean follow-up: 64.75 months (9–124 months). All 4 cases had excellent/good MSTS scores (25–29 points). Imaging showed satisfactory bone graft healing; no AVN, recurrence, or limp/pain occurred.

**Conclusion:**

Supine DAA-SHD (no trochanteric osteotomy) is effective for adolescent femoral head chondroblastoma, enabling complete resection, anatomical reconstruction, and vascular protection. It enriches pediatric hip tumor treatment options but needs validation via large-scale prospective studies.

## 1 Introduction

**Figure 1:**
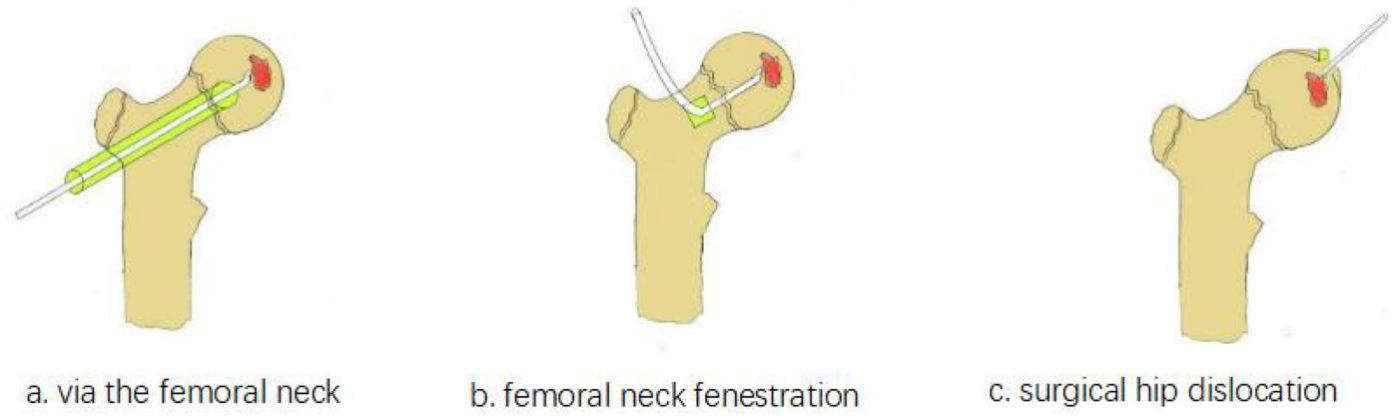
**(a-c)** planning diagrams of the three surgical approaches for chondroblastoma of the femoral neck.

**Figure 2:**
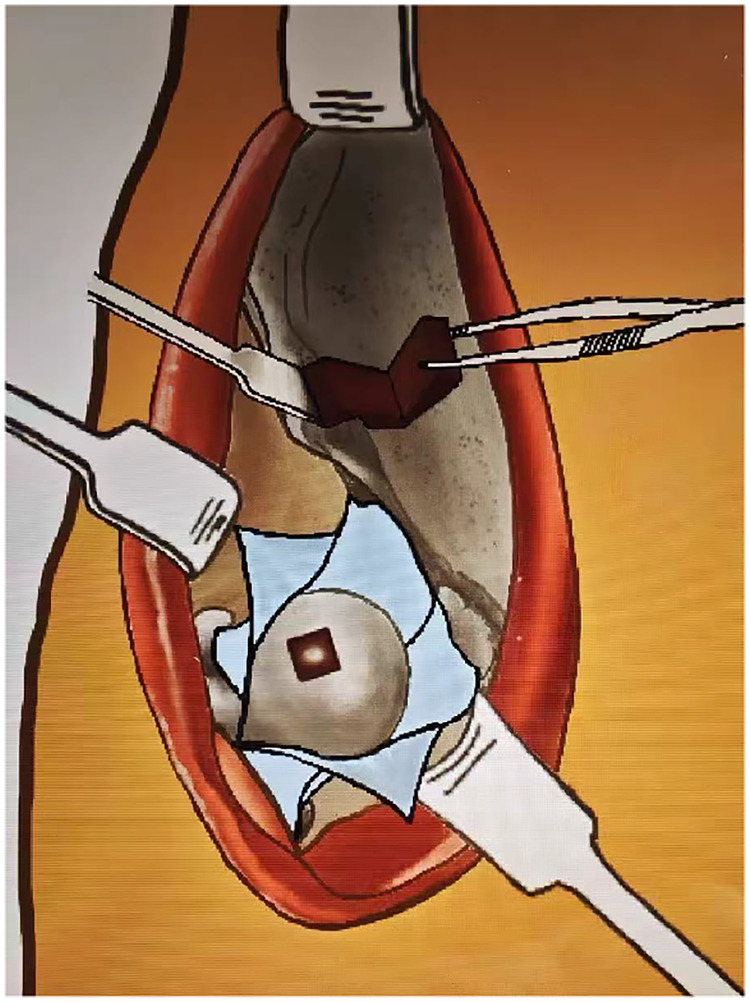
Schematic diagram of the DAA-SHD procedure: tumor resection, bone grafting, autologous iliac bone harvesting, and articular repair via a single incision in the supine position.

**Figure 3:**
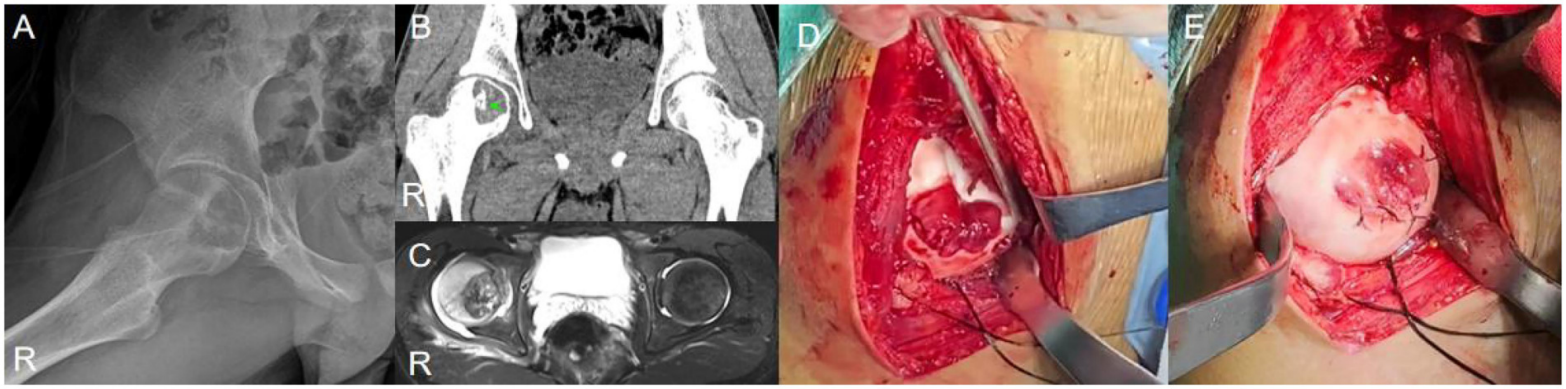
Case 1: **(A)** preoperative x-ray showed an isolated eccentric osteolytic lesion in the right femoral head. **(B)** CT (soft tissue window): Calcified tissue visible (green arrow). **(C)** MRI (T2WI-STIR): High signal in the femoral head on the tumor side, with irregular signals within the tumor. **(D)** Dislocation revealed joint cartilage destruction and joint surface collapse and deformity. **(E)** The smooth autologous iliac cortical bone was used for femoral head osteoplasty, and the damaged articular cartilage was sutured and repaired to flatten the joint surface.

Chondroblastoma, first delineated by Jaffe and Lichtenstein in 1942, is a rare benign bone tumor accounting for approximately 1%–2% of all bone neoplasms and 9% of benign bone tumors (1, 2). It typically arises in the epiphyses or apophyses of long bones in adolescents and young adults, with a male predominance (2:1 ratio). The femoral head represents an uncommon yet challenging site due to its intra-articular position and critical vascular anatomy. The Direct Anterior Approach (DAA), refined by pioneers such as Smith-Petersen and Judet, utilizes internervous and intermuscular planes to access the hip joint (3, 4). Its application in hip arthroplasty by Matta further established its utility in preserving periarticular soft tissues and minimizing vascular disruption (5). Concurrently, the Surgical Hip Dislocation (SHD) technique, pioneered by Ganz et al., was developed based on detailed vascular studies to allow 360° visualization of the femoral head while safeguarding its blood supply, traditionally involving a trochanteric osteotomy (6). The pathobiology of chondroblastoma has been increasingly elucidated, with mutations in genes encoding histone H3.3 (H3F3B) being implicated in a majority of cases (7, 8). Clinical presentation often includes insidious hip or groin pain, sometimes exacerbated by activity and poorly responsive to analgesics (9). Although benign, chondroblastomas can exhibit aggressive local behavior, and rare instances of pulmonary metastasis or malignant transformation have been documented (10–13). Current therapeutic paradigms emphasize complete tumor excision to mitigate recurrence. Standard surgical options include: (1) minimally invasive curettage via a femoral neck tunnel (Figure 1A), (2) an anterior approach with a femoral neck cortical window (Figure 1B), and (3) surgical hip dislocation for direct articular access (14–18) (Figure 1C). Each technique carries distinct trade-offs regarding exposure completeness, articular cartilage preservation, and risk to the femoral head vasculature and physis. We summarized case reports on the surgical management of femoral head chondroblastoma published over the past two decades (2005–2025) (Table 1) and present a case series in which four adolescent patients with femoral head chondroblastoma underwent tumor resection and articular reconstruction using the Direct Anterior Approach combined with Surgical Hip Dislocation (DAA-SHD) technique, performed in the supine position without trochanteric osteotomy. Additionally, we summarize the clinical features, diagnosis, and treatment of femoral head chondroblastoma, and discuss our clinical experience with this condition.

**Table 1:** Case report on surgical management and efficacy of chondroblastoma of the femoral head.

Author	Year	Number of cases	Patient position	Surgical procedure	Outcome
Di Yang	2024	6 CB	30-degree angle relative to the operating table.	Without Surgical Dislocation + absorbable cartilage pins fixation	1 Femoral Neck Fracture
Qichao Ma	2024	2 LCH, 2 CB, 1 Uncertain	Supine position	Surgical Dislocation + Trapdoor Procedure + Smith-Petersen + absorbable sutures	2 LCH AVN, 1 LLD, FAI, 2 CB no complications
Gersh MP	2023	1 CB	Supine position	Surgical Dislocation + X-REAM minimally invasive approach	LLD
Hirohisa Katagiri	2022	2 CB	Semi-lateral position	Surgical Dislocation + Trapdoor Procedure	no AVN
Mohamed Abo-Elsoud	2021	10 CB	Lateral position	Surgical Dislocation + Ganz osteotomy + modified trapdoor	1 recurrence, 2 flexion deformity, 1 heterotopic ossification
Tarun Verma	2018	1 CB	Lateral position	Surgical Dislocation + Ganz osteotomy + mosaicplasty	No complications within 2 years
Liu Q	2019	17 CB	Lateral position	Surgical Dislocation + Ganz osteotomy + modified trapdoor	1 AVN, 1 OA
Orlando-Díaz C	2014	1 CB	/	Surgical Dislocation + humeral cartilage transplantation fixation	No complications within 3 years
Hairong Xu MD	2014	13 CB	Supine position	Smith-Petersen Surgical Dislocation + Modified Trapdoor Procedure	1 AVN, 1 heterotopic ossification
Panagiotis GiviSSiS	2012	1 CB	Lateral position	Hardinge Surgical Dislocation	No complications within 2 years
BARTH RIEDEL, MD	2012	1 CB	Lateral position	Vascularized fi bula transfer	Joint degeneration after 9 years
Stefano Stilli	2010	1 CB	Supine position	Smith-Petersen Surgical Dislocation + a frozen femoral head transplantation	LLD occurred at 2 years, joint degeneration occurred at 7.5 years
Toshiya Iwai	2007	1 CB	Lateral position	Surgical Dislocation + trapdoor procedure	No complications within 5 years
Maezawa K	2005	1 CB	Supine position	Greater trochanteric windowing + Rotational Acetabular Osteotomy	No complications within 4.5 years

CB, chondroblastoma; LCH, langerhans cell histiocytosis; AVN, avascular necrosis; LLD, leg length discrepancy; OA, osteoarthritis.

## 2 The DAA-SHD approach

The DAA-SHD procedure is performed with the patient supine. An 8–10 cm incision is made from the anterior superior iliac spine towards the anterior aspect of the greater trochanter (Figure 2). The Hueter interval between the tensor fasciae latae and sartorius is developed, with care to protect the lateral femoral cutaneous nerve. The lateral femoral circumflex vessels are identified, ligated, and divided. The interval between the rectus femoris (whose reflected head is detached and tagged for later repair) and gluteus medius is entered, exposing the anterior hip capsule.

A T-shaped capsulotomy is performed, meticulously preserving the retinacular vessels along the femoral neck. Under gentle traction and external rotation, the ligamentum teres is transected, permitting anterior hip dislocation. This maneuver provides circumferential access to the femoral head.

Using preoperative imaging and sometimes a 3D-printed model for guidance, a small cartilage window is created directly over the tumor, avoiding the primary weight-bearing dome. The lesion is meticulously curetted, and the sclerotic rim is burred until viable, bleeding bone is encountered. The cavity is irrigated with sterile water and ethanol for adjuvant effect. The resultant defect is grafted using a combination of autologous iliac cancellous bone, allograft chips, calcium sulfate, and osteoinductive materials. For cartilage defects, a contoured autologous iliac cortical bone graft is used to reconstruct the articular surface, secured with absorbable sutures. Following reduction, the capsule and rectus femoris are repaired anatomically. Postoperative management entails a period of non-weight-bearing to protect the reconstruction.

## 3 Literature review and comparative analysis

Management of femoral head chondroblastoma must balance complete tumor eradication against the preservation of hip function and vascular integrity. The following surgical approaches are documented in the literature:

### 3.1 Femoral neck tunnel approach

This minimally invasive technique involves creating a bone tunnel from the lateral cortex, below the greater trochanter, towards the femoral head lesion (15). While minimizing soft tissue dissection, it offers limited visualization, potentially leading to incomplete tumor removal and elevated recurrence risk (19, 20). The confined working space and proximity to the physis are additional limitations.

### 3.2 Anterior femoral neck cortical window approach

This method provides better exposure than the tunnel technique but still does not allow direct visualization of the articular surface (16). Creating a window in the femoral neck risks injuring the physis and the ascending cervical arteries, potentially leading to AVN or femoral neck fracture. It does not permit repair of damaged articular cartilage.

### 3.3 Surgical hip dislocation with trochanteric osteotomy

The Ganz SHD provides excellent exposure of the entire femoral head and acetabulum, enabling direct tumor resection and cartilage repair (6, 17, 18). The principal concern has been the potential for AVN and complications related to trochanteric osteotomy healing, such as non-union or hardware irritation.

The DAA-SHD approach, as described in our series, integrates the advantages of the classic SHD—direct visualization and articular access—with the soft-tissue-preserving benefits of the DAA. By avoiding trochanteric osteotomy and utilizing a supine position, it potentially reduces the risk of AVN, facilitates intraoperative imaging, and may simplify the procedure.

## 4 Case presentation

Preoperatively, plain x-rays consistently revealed features of benign epiphyseal cartilaginous tumors, including isolated eccentric osteolytic changes that were adjacent to or had penetrated the physeal plate, thinning of the cortical bone, well-defined margins, which might be accompanied by sclerotic rims, and a small amount of intralesional calcifications (these features are more prominent on CT) (21, 22). These findings differ from the typical “soap bubble-like” changes of juvenile giant cell tumors of bone, which generally lack calcifications (23–25). Differential diagnosis should be made with bone-derived tumors and osteoblastomas (which are commonly located in the spine) (26, 27). Additionally, distinction should be drawn from enchondromas, which are characterized by a typical “ring-and-arc” intralesional calcification pattern and expansile osteolytic lesions (28–30). For cases with atypical x-ray findings, further CT and MRI examinations are recommended. All underwent tumor curettage, bone grafting, and autologous iliac cortical bone-supported articular reconstruction via DAA-SHD.

### 4.1 Case 1

A 13-year-old male, ethnicity: Han, presented with right hip pain and discomfort without any obvious cause for 1 year, with a worsening of symptoms over the past 2 months (Figure 3). The pain was mostly continuous, worsening at night. Initially, the patient self-medicated with nonsteroidal anti-inflammatory drugs (NSAIDs) with some relief, but in the last 2 months, the pain worsened, and the pain relief from NSAIDs became ineffective. Physical examination revealed tenderness at the midpoint of the right inguinal region, a positive 4-sign test. Both lower limbs were of equal length.

### 4.2 Case 2

**Figure 4:**
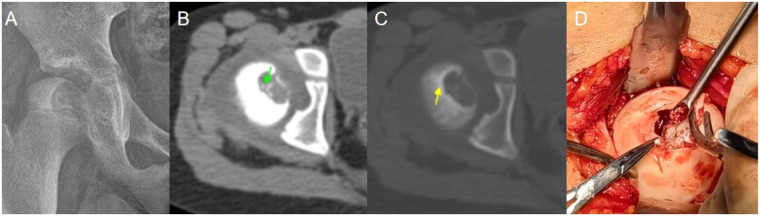
Case 2: **(A)** preoperative x-ray showed an eccentric osteolytic lesion in the medial column, involving the center of the femoral head. **(B)** CT (soft tissue window): Irregular calcified tissue was seen within the tumor (green arrow). **(C)** CT (bone window): Sclerotic margins were present around the lesion (yellow arrow). **(D)** Intraoperatively, pathological destruction of the articular cartilage and collapse of the femoral head were observed; the lesion was completely curetted under direct visualization, and autologous iliac bone graft was harvested for reconstruction to support the articular surface.

A 9-year-old girl, ethnicity: Zhuang, presented with right hip pain and discomfort for 3 months following a collision (Figure 4). The pain was intermittent, worsening after activity. Her gait was normal, but she had a positive 4-sign test on the right side, with increased pain on hip hyperextension.

### 4.3 Case 3

**Figure 5:**
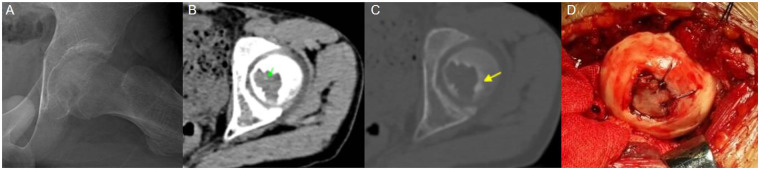
Case 3: **(A)** x-ray: a lucent low-density lesion is present in the medial aspect of the left femoral head. **(B)** CT (soft tissue window): Punctate calcifications (green arrow). **(C)** CT (bone window): a thin sclerotic margin surrounds the lesion.(yellow arrow). **(D)** The smooth cortical surface of the iliac bone was used to suture and repair the necrotic articular cartilage surface.

A 12-year-old male, ethnicity: Han, presented with left hip pain for more than 1 year (Figure 5). The pain, located in the groin area, appeared without any obvious cause and was significantly aggravated by intense activity. Physical examination revealed tenderness at the left groin and mid-thigh, with a positive 4-sign test.

### 4.4 Case 4

**Figure 6:**
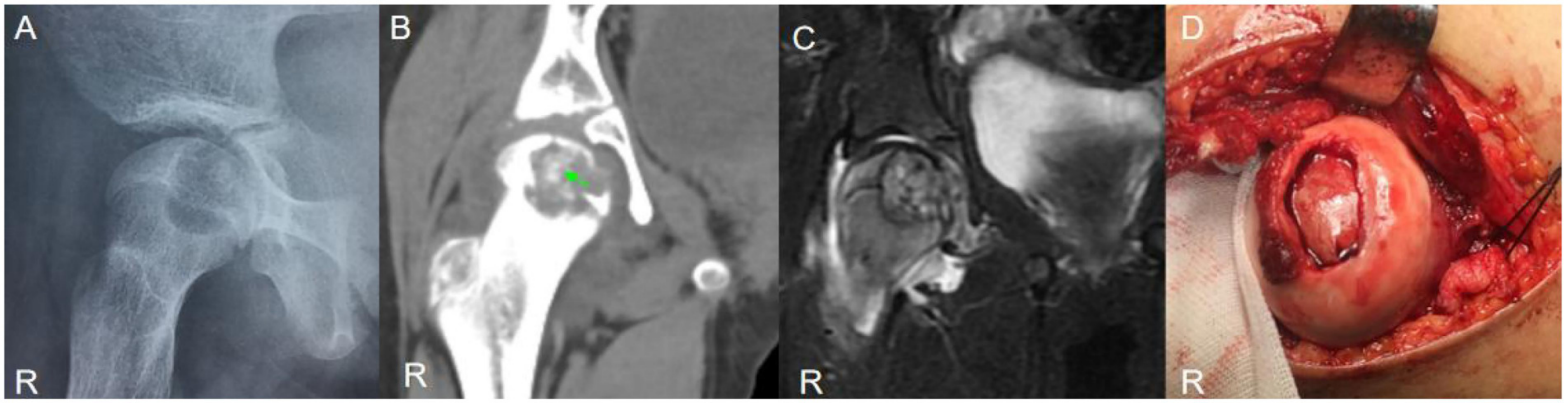
Case 4: **(A)** an isolated eccentric osteolytic lesion in the right femoral head. **(B)** CT (soft tissue window): Irregular calcified tissue (green arrow). **(C)** MRI (T2WI-STIR): high signal in the femoral head on the tumor side, with irregular signals within the tumor. **(D)** After curetting the tumor tissue under direct visualization, autologous iliac bone was harvested for grafting to support the cavity and repair the damaged articular surface.

A 12-year-old female, ethnicity: Zhuang, presented with right hip pain for more than 7 months without any obvious cause (Figure 6). The pain worsened with walking, and the patient had a limping gait. Tenderness was noted at the midpoint of the right groin. A x-ray imaging suggested Chondroblastomas.

## 5 Results

**Figure 7:**
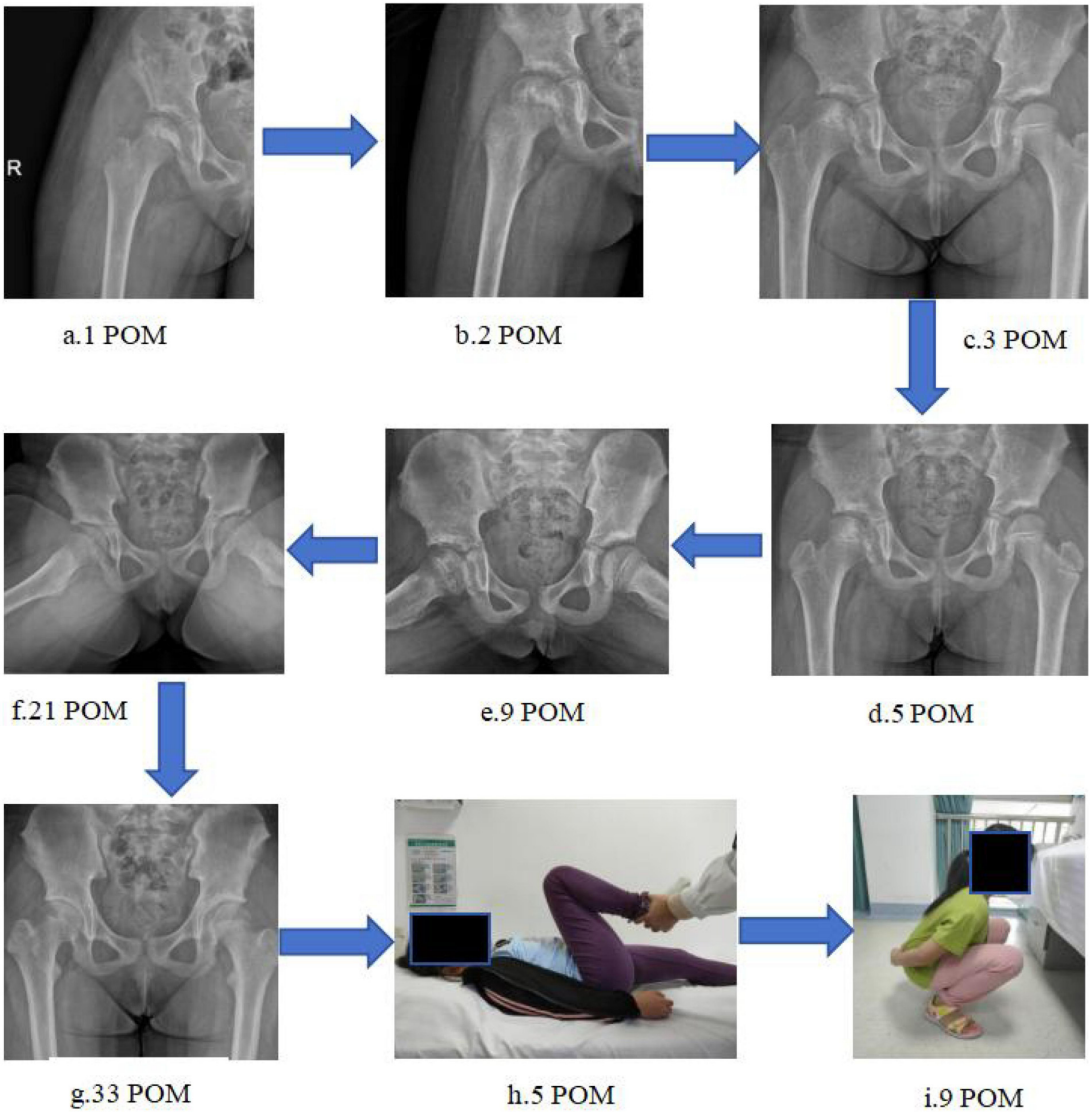
**(a–i)** Illustrate the postoperative follow-up process of case 2. The patient received ankle pump exercises within 1 week, knee flexion-extension exercises within 2 weeks, and gradual hip flexion training within 3 weeks, then progressive weight-bearing training. No hip surgery-related complications were observed, including arthralgia, joint stiffness, and femoral head necrosis. **(h)** Physical examination at 5 months postoperatively showed no significant limitation in hip joint range of motion; **(i)** At 9 months postoperatively, the patient had returned to normal daily activities.

The mean postoperative follow-up was 64.75 months (range: 9–124 months). The Musculoskeletal Tumor Society (MSTS) scale, a validated tool for evaluating post-treatment limb function in bone tumor patients, includes 6 items: pain, functional activity, emotional acceptance, walking ability, gait, and use of Support devices, with a total score of 30 (excellent: 27–30; good: 24–26; fair: 20–23; poor: <20) (31). In this study, all patients achieved excellent functional outcomes, with MSTS scores ranging from 25 to 29 (mean: 27.75, Table 2). Detailed items of the MSTS scale and interpretation of score grades are presented in Table 3 (see footnote a in Table 3). Follow-up imaging demonstrated satisfactory bone graft incorporation, maintenance of femoral head sphericity, and no evidence of AVN, joint degeneration, leg length discrepancy, or tumor recurrence. All patients resumed normal activities without pain or gait abnormality. We presented the follow-up data of one of the patients (Figure 7).

**Table 2:** General information statistical table.

Case information	Value
No. of patients	4
Sex	
Male	2 (50%)
Female	2 (50%)
Operative side	
Left	1 (25%)
Right	3 (75%)
Age at diagnosis (years)	
Mean	11.5
Range	9–13
Follow-up time (mths)	
Mean	64.75
Range	9–124
MSTS scores^a^	
27–30 (excellent)	3 (75%)
24–26 (good)	1 (25%)
21–23 (fair)	0
20≤ (poor)	0

a The items are listed in Table 3.

**Table 3:** The musculoskeletal tumor society scale.

Case	Pain	Function	Emotional acceptance	Supports	Walking	Gait	Total
1	4	4	5	4	4	4	25
2	5	5	5	5	4	5	29
3	4	4	5	5	5	5	28
4	4	5	5	5	5	5	29

a The MSTS items.

**Figure 8:**
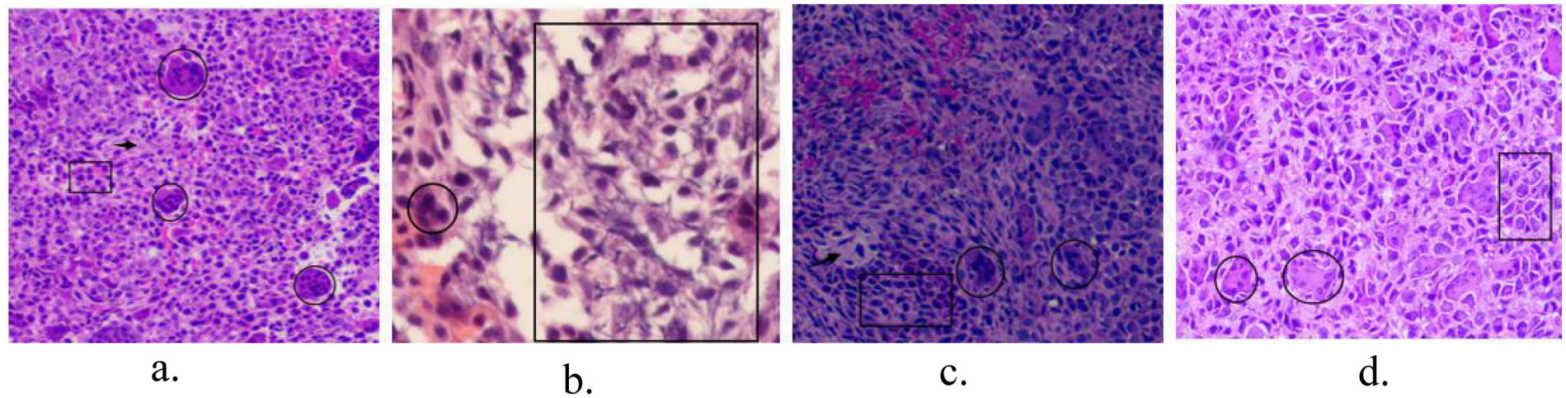
**(a–d)** Pathological tissues from 4 different cases postoperatively, showing chondroblasts (rectangles), surrounded by chondroid matrix, with partial calcification (arrow in **c**), scattered multinucleated giant cells (circles). **(b)** Characteristic pericellular chicken wire-type calcification (rectangle) [HE; **(a,c,d)** 4 × 10; **(b)** 10 × 10; immunohistochemistry: **(c)** S-100 (−)].

As early as 1972, David C. Dahlin, John C. Ivins based on their pathological studies of 125 case ts, Andrew G. Huvos published pathological findings of 25 chondroblastoma cases in Cancer, proposedhat chondroblastoma is pathologically characterized by the presence of osteoclast-like multinucleated giant cells, cobblestone-like mononuclear oval chondroblasts, eosinophilic chondroid matrix, partial visible calcified tissue, and lattice-like or Pericellular chicken wire-type calcification (32, 33). Later, studies by G. Edel (53 cases), Chandu de Silva (42 cases) and other researchers confirmed these pathological changes, and also verified that the positive expression of S-100 protein in immunohistochemistry can specifically assist in the diagnosis of chondroblastoma (34, 35). We observed these pathological changes in the postoperative pathological tissue sections. Among the immunohistochemical results of the four patients, although the S-100 protein expression was negative in Case 3, its pathological changes were consistent with the diagnosis of chondroblastoma (Figure 8).

## 6 Discussion

The management of femoral head chondroblastoma in children is fraught with the dual challenges of ensuring oncological control and preserving hip development and function. The DAA-SHD approach addresses several limitations of conventional techniques. Oncological Adequacy: Direct visualization ensures complete tumor removal, theoretically reducing recurrence risk compared to blind or fluoroscopically-assisted curettage. Vascular Safety: The DAA respects the posterior vascular structures, primarily the medial femoral circumflex artery, which is the dominant blood supply to the femoral head (36, 37). The avoidance of trochanteric osteotomy further minimizes vascular insult. Articular Surface Restoration: A unique advantage of this approach is the ability to directly address articular cartilage damage. The use of a supportive iliac cortical bone graft to reconstruct the subchondral bone and cover cartilage defects is a key technical nuance that may help prevent joint collapse and post-traumatic arthritis (38). Physeal Preservation: By accessing the tumor directly through the articular surface, the DAA-SHD approach avoids creating a path through the femoral neck, thereby minimizing iatrogenic injury to the physis and reducing the risk of growth disturbance. While the Ganz SHD is a proven technique, the DAA-SHD variant offers a logical alternative, particularly for anteriorly located femoral head pathologies. The supine position aids in orientation and allows for concurrent bilateral hip imaging. The learning curve for this procedure is notable, requiring expertise in hip preservation surgery (39, 40). Comparisons with recently described “trapdoor” techniques without dislocation highlight a strategic difference (41). The DAA-SHD approach intentionally dislocates the hip to gain unrestricted access, which is crucial when the tumor has eroded or breached the cartilage, necessitating direct repair. This controlled dislocation is performed with meticulous attention to vascular preservation, making it a safe maneuver in experienced hands.

## 7 Limitation

The conclusions drawn are constrained by the inherent limitations of a small, single-center case series. The favorable outcomes require validation through larger, prospective studies with long-term follow-up to definitively establish the procedure's efficacy in preventing AVN and osteoarthritis. The technical demands and associated learning curve may limit its widespread adoption.

## 8 Conclusions

The supine DAA-SHD approach without trochanteric osteotomy represents a sophisticated and effective surgical strategy for managing chondroblastoma of the femoral head in adolescents. By combining the principles of the DAA and SHD, it facilitates complete tumor excision under direct vision, enables anatomical reconstruction of the articular surface, and minimizes the risk of vascular compromise. This review and case series contribute to the growing body of evidence supporting this technique as a valuable option in the armamentarium for treating complex pediatric hip tumors, warranting further comparative investigation. It is also expected to provide new insights into the surgical treatment of other types of femoral head tumors.

## Data Availability

The original contributions presented in the study are included in the article/Supplementary Material, further inquiries can be directed to the corresponding authors.
