# Supplementary material for: Case Report: Direct anterior approach with surgical hip dislocation for management of juvenile femoral head chondroblastoma: a case series and systematic review of the literature
Source: Front Pediatr. 2026 Jan 21;13:1738552. doi: 10.3389/fped.2025.1738552 (PMC12868215; doi:10.3389/fped.2025.1738552)
Supplement: Supplementary file 1 [file Datasheet1.pdf]

CARE Checklist of information to include when writing a case report

CARE case report guidelines

(cc) BY-NC-ND

Topic	Item	Checklist item description	Reported on Line
Title	1	The diagnosis or intervention of primary focus followed by the words "case report"	title
Key Words	2	2 to 5 key words that identify diagnoses or interventions in this case report, including "case report"	key words
Abstract (no references)	3a	Introduction: What is unique about this case and what does it add to the scientific literature?	Introduction
	3b	Main symptoms and/or important clinical findings	Case presentatior
	3c	The main diagnoses, therapeutic interventions, and outcomes	Case presentation
	3d	Conclusion—What is the main "take-away" lesson(s) from this case?	conclusion
Introduction	4	One or two paragraphs summarizing why this case is unique (**may include references**)	Case presentation
Patient Information	5a	De-identified patient specific information.	Introduction
	5b	Primary concerns and symptoms of the patient.	Case presentation
	5c	Medical, family, and psycho-social history including relevant genetic information	Case presentatior
	5d	Relevant past interventions with outcomes	Case presentation
Clinical Findings	6	Describe significant physical examination (PE) and important clinical findings.	Case presentatior
Timeline	7	Historical and current information from this episode of care organized as a timeline	Case presentatior
Diagnostic Assessment	8a	Diagnostic testing (such as PE, laboratory testing, imaging, surveys).	Case presentatior
	8b	Diagnostic challenges (such as access to testing, financial, or cultural)	Case presentatior
	8c	Diagnosis (including other diagnoses considered)	Result
	8d	Prognosis (such as staging in oncology) where applicable	Result
Therapeutic Intervention	9a	Types of therapeutic intervention (such as pharmacologic, surgical, preventive, self-care)	The DAA-SHD Approach
	9b	Administration of therapeutic intervention (such as dosage, strength, duration)	The DAA-SHD Approac
	9c	Changes in therapeutic intervention (with rationale)	The DAA-SHD Approc
Follow-up and Outcomes	10a	Clinician and patient-assessed outcomes (if available)	Result
	10b	Important follow-up diagnostic and other test results	Result
	10c	Intervention adherence and tolerability (How was this assessed?)	Literature Review
	10d	Adverse and unanticipated events	no
Discussion	11a	A scientific discussion of the strengths AND limitations associated with this case report	Discussion
	11b	Discussion of the relevant medical literature **with references.**	Discussion
	11c	The scientific rationale for any conclusions (including assessment of possible causes)	Discussion
	11d	The primary "take-away" lessons of this case report (without references) in a one paragraph conclusion	Conclusions
Patient Perspective	12	The patient should share their perspective in one to two paragraphs on the treatment(s) they received	Discussion
Informed Consent	13	Did the patient give informed consent? Please provide if requested	Yes ☑ No ☐
